# Decomposition Characteristics of SF_6_ under Flashover Discharge on the Epoxy Resin Surface

**DOI:** 10.3390/ma12091408

**Published:** 2019-04-30

**Authors:** Hao Wen, Xiaoxing Zhang, Rong Xia, Guoxiong Hu, Yunjian Wu

**Affiliations:** 1 School of Electrical Engineering, Wuhan University, Wuhan 430072, China; wenhao198711@163.com (H.W.); 15733221344@163.com (G.H.); wuyunjian@whu.edu.cn (Y.W.); 2 State Grid Electric Power Research Institute, Wuhan Nari Co Ltd, Wuhan 430074, China; 3 Wuhan Branch, China Electric Power Research Institute Co., Ltd., Wuhan 430074, China; xiarong@epri.sgcc.com.cn

**Keywords:** flashover discharge, SF_6_ decomposition, epoxy resin, characteristic gases

## Abstract

In this paper, the flashover discharging experiment was carried out on epoxy resin surface in an SF_6_ atmosphere under pin-plate electrodes, with the electrodes distance from 5 mm to 9 mm. The concentration of seven characteristic gases was detected, indicating that the concentration of SOF_2_ and CF_4_ was the two highest, followed by SO_2_, CO_2_, SO_2_F_2_, CS_2_, and H_2_S. Based on the changes in the concentration of the characteristic gases, a preliminary rule was proposed to predict the occurrence of flashover discharge on epoxy resin: When the concentration of SOF_2_ reaches twice of CF_4_ concentration, and the total concentration of both SOF_2_ and CF_4_ is much higher than that of H_2_S, a possible flashover discharge on the epoxy resin surface in SF_6_-infused electrical equipment occurs. Through the simulation of decomposition of epoxy resin, it has been revealed that H_2_O has different generation paths that can facilitate the formation of SOF_2_, finally influencing the concentration variation of the seven characteristic gases.

## 1. Introduction

Gas-insulated electrical equipment has been widely used in power system because of their advantages such as compact size, high reliability, and a long maintenance period [1,2]. It was found that a considerable proportion of the internal faults of GIS (gas insulated switchgear) are closely related to the insulating spacer used to support the copper conductor, among which the surface discharge of insulating spacer is hard to be detected due to its randomness and instantaneity [3,4]. So far, attention has been paid to the fault detection technology for insulating spacer. The monitoring system on the decomposed gases of SF_6_ develops rapidly, which provides an effective way to judge the operation situation of SF_6_- infused electrical equipment. 

SF_6_ can decompose into a series of subfluorides and sulfides when high energy imposes on SF_6_ molecules on the condition of partial discharge, overheating, corona, electrical breakdown, or even ultraviolet radiation [5,6,7]. Conventionally, subfluorides can recombine with fluorinion to form SF_6_ molecules again, owing to the intrinsic electronegativity property of SF_6_. However, in most cases, air and water vapor mixed with SF_6_ exists. These impurities react with subfluorides and produce various gases like SOF_4_, SOF_2_, SO_2_F_2_, SF_4_, SO_2_, CF_4_, CO_2_, H_2_S, and so on [8,9,10], which prevent the recombination of SF_6_ and subsequently reduce its insulation strength. Based on the concentration variation, a preliminary fault diagnosis method was provided to judge running situation of electrical equipment [8,9,10]. However, given the complicated internal situation of electrical equipment, there are many influential factors that tend to make concentration of decomposition components from SF_6_ fluctuate drastically, such as temperature, humidity, and running voltage. Flashover discharge on the surface of epoxy resin gives rise to partial decomposition of epoxy resin, leading to potential chemical reaction between epoxy resin and SF_6_ decomposition byproducts. However, this critical influential factor on SF_6_ byproduct concentration attracts less attention today.

The decomposition of SF_6_ has been extensively studied. Sausers et al. [11] conducted experiments on the effect of oxygen on SF_6_ decomposition byproducts and found that oxygen can promote the production of SO_2_F_2_ and SOF_4_, which has little effect on the generation rate of SOF_2_. Derdouri et al. [12] designed a study on the SF_6_ decomposition products when water vapor exists and found that SOF_2_ and SO_2_F_2_ generate stably in existence of water vapor. Later, it was found that SF_6_ decomposition byproducts, including SOF_4_, SOF_2_, SO_2_F_2_, SO_2_, HF, would appear as long as water vapor and oxygen existed [13]. Additionally, a study reported that several SF_6_ decomposition byproducts under four different kinds of partial discharge models, showing that CF_4_, CO_2_, SO_2_F_2_, SOF_4_, SOF_2_, SO_2_, and H_2_S were main decomposition products of SF_6_, among which the content of SOF_2_ and SO_2_F_2_ were the highest [14]. Furthermore, Belmadani et al. [15] explored the characteristics of SF_6_ decomposition products under AC arc and pointed out that the total content of SOF_2_ and SO_2_ would increase with current intensity. It has been generally accepted that all SF_6_ decomposition products concentration would increase with discharge energy, regardless of the kind of discharge [16,17,18]. The influences of solid insulation material on the decomposition of SF_6_ were also studied. For example, it was reported that CF_4_ would appear [12], since organic materials contain abundant C-H bonds that can easily react with fluorinion under high-energy arc discharge. Afterward, a small amount of CS_2_ was detected when flashover discharges happened on epoxy resin surface in 110 kV GIS, thus making it a characteristic gas in a fault detection system [19]. There is no dispute that SOF_2_, SO_2_F_2_, and SO_2_ will occur easily in partial discharge situation, and that H_2_S would be generated when discharge energy is high enough. Moreover, CF_4_, CO_2_, and CS_2_ would be generated when organic solid insulation materials decomposed partially under high voltage [12,15].

So far, no report has focused on how epoxy resin affects the decomposition of SF_6_ under flashover discharge yet. Additionally, it is too complicated to elaborate the chemical mechanism behind it. Although thermal and mechanical of epoxy resin can be enhanced through incorporating some filler into the polymer matrix, it still can decompose under arc discharge [20,21,22]. In the current study, pin-plate electrodes on the epoxy resin insulation spacer surface were designed; flashover discharge at different distances of pin-plate electrodes was carried out; and the concentration of decomposition byproducts of SF_6_, including SOF_2_, SO_2_F_2_, SO_2_, H_2_S, CF_4_, CO_2_, and CS_2_, was examined in order to find the relationship between decomposition of SF_6_ under flashover discharge on the epoxy resin surface. Besides, by simulating decomposition process of epoxy resin, the generation path of each gas was revealed.

## 2. Materials and Methods

### 2.1. Materials

In the present study, the bisphenol-A epoxy resin E51 (DGEBA) was purchased from Wuxi Lan-Star Petrochemical Co., Ltd. (Wuxi, China). Epoxy resin was cured by an amine curing agent consisting of an adduct of diethylenetriamine and butyl glycidyl ether, known as 593 curing agent, supplied by Wuhan Shen Chemical Reagents and Equipments Co., Ltd. (Wuhan, China). High-precision SF_6_ gas with grade of purity of 99.999% was supplied by Wuhan Newradar Gas Co., Ltd. (Wuhan, China). Therefore, the original percentage content of O_2_ and H_2_O in the gas was low enough to be omitted.

### 2.2. Experiment Platform

Among a multitude of documented electrical faults, flashover in the electrical equipment happens frequently. Today flashover fault can be detected easily, but it is still hard to explain the complicated mechanism behind it [5,6,7]. Flashover discharge emphasizes on electrical breakdown of gap happening on solid material surface, such as flashover discharge on basin spacer surface in gas-insulated electrical equipment, where flashover voltage or flashover current was studied. Therefore, in our experiment, pin-plate electrodes on the epoxy resin surface was devised to investigate influence of surface breakdown of epoxy resin on SF_6_ decomposition byproducts category and their concentration.

**Figure 1:**
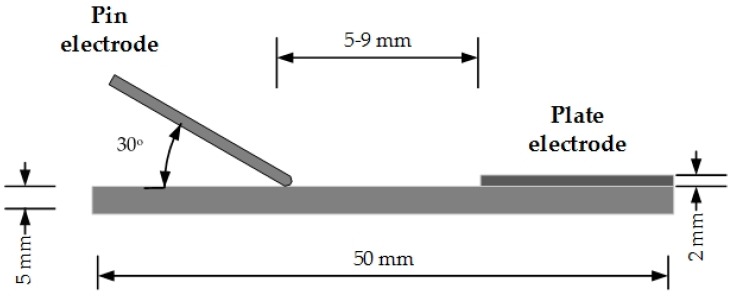
Pin-plate electrodes on epoxy resin.

**Figure 2:**
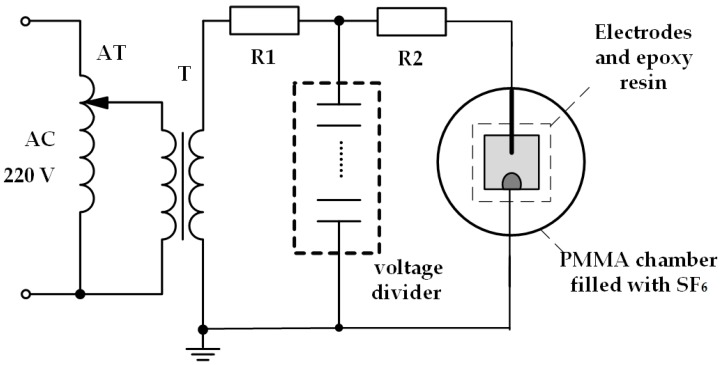
AC experimental equipment.

To create a severely uneven electrical field, pin-plate electrodes were used in this study, as shown in Figure 1. Given that the conducting rod in high-voltage cable is made of copper with better electric conductivity, the positive electrode in our study was a long-customized needle made of copper, the curvature radius of the needle electrode is 0.5 mm and beveled at 30° on the sample surface. The plate electrode is a semicircle copper sheet with a diameter of 10 mm and is stuck to the surface of epoxy resin with a small amount of conductive silver paste to ensure that the electrical arc was close enough to the surface when the gap breaks down. The epoxy spacer sample is a square piece with a size of 50 mm × 50 mm × 2 mm. The pin-plate electrodes are positioned in a sealed cylinder made of polymethyl methacrylate (PMMA) as shown in Figure 2. The AC high voltage power can provide a maximum value of 50 kV. Concentration of decomposed components of SF_6_ was measured on the gas chromatograph-mass spectrometer (GC-MS) and the type of GC-MS is Shimadzu QP-2010Ultra (Shimadzu Co., LTD, Kyoto, Japan).

To protect the voltage divider, the value of R2 in Figure 2 was usually below 200 Ω, and the value of R1 was usually above 3000 Ω to protect the transformer when voltage divider was totally destroyed. Because epoxy resin has a high electrical resistivity with an order of magnitude above 10^13^ Ω cm, it is fully accepted that the break-down voltage on the sample surface equals to the highest shown value on the operating board when the gap was not broken down.

Because roughness of sample surface has a significant influence on flashover discharge property of epoxy resin, roughness of the samples was assessed on the Surface Profilometer 2300 supplied by Wale Electromechanical Technology Co., Ltd. (Xi’an, China). Roughness of all the epoxy resin samples had an average value of 0.35 μm in the range from 0.27 μm to 0.48 μm. Conventionally, roughness of solid material surface should be kept below 1 μm in order to ensure the AC (Alternating Current) arc creeping closely on the sample surface.

### 2.3. Experiment Procedures

The specific experimental procedures are listed as follows:

First, all experiment materials were cleaned with anhydrous ethanol to get rid of dust and other impurities that may adhere to the experiment materials. After being volatilized with anhydrous ethanol, the epoxy spacer and electrodes were placed in the fixed position in the container. The distance between the pin-plate electrodes was set at 5, 6, 7, 8 and 9 mm, respectively. Then, the container was sealed.

Second, the chamber was evacuated to 0.01 MPa and filled with fresh SF_6_ until the pressure reached 0.2 MPa. It needed to take three times to remove most of water vapor and air. Subsequently, 100 mL of gas was extracted as the initial reference. 

Finally, voltage was imposed slowly at the rate of 2 kV/30 s until the gap between the electrodes broke down, then the discharge voltage was recorded. After the decomposed gases diffused for 3 min in the chamber, 100 mL of gas was extracted from the chamber and then injected into the GC-MS. Twenty-one flashover discharge times were conducted for each gap distance in order to find the basic growth law of SF_6_ decomposition byproducts. As we all know, the surface flashover does significant harm to the dielectric property of epoxy resin. As the discharge number increased, the flashover voltage decreased slowly. The small decomposition, or the carbonization of the epoxy resin surface can cause obvious characteristic gases concentration change [23]. Therefore, 21 times of flashover discharges are enough to make significant harm to dielectric properties of epoxy resin and create more precise concentration variation.

## 3. Results and Discussion

### 3.1. Discharge Voltages Comparison

**Figure 3:**
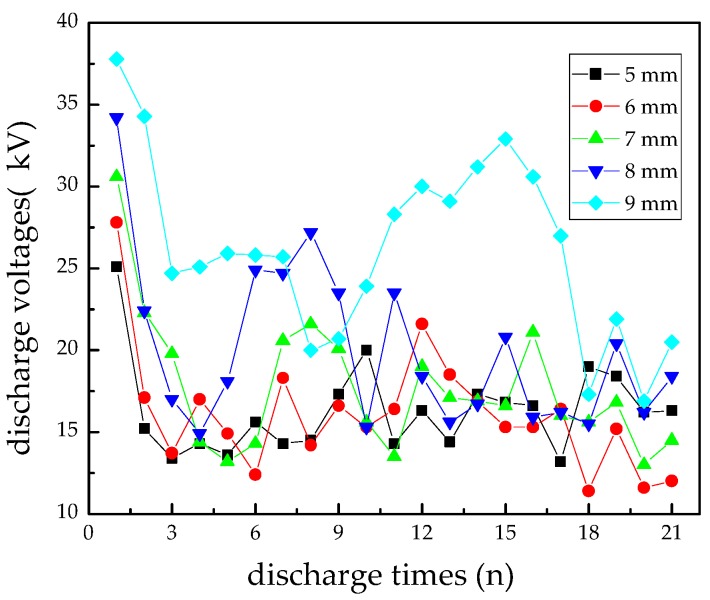
Initial flashover discharge voltages vs. discharge times at different pin-plate electrodes distances.

Figure 3 shows initial flashover discharge voltages vs. discharge times at different pin-plate electrodes distances. It is apparent that discharge voltage decreased rapidly in the first three times flashover discharges. Then, discharge voltage fluctuated slowly in a downward trend on the whole. After the breakdown of the gap between the electrodes, roughness of the epoxy resin sample surface increased with an obvious carbonization trace, which would greatly reduce flashover discharge voltage under the uneven electrical field. At the same time, water vapor generating during the decomposition of epoxy resin could also enhance the conductivity of carbonized trace on the surface [24]. As a result, the flashover discharge voltage decreased in the first three times. However, the roughness of the sample surface did not markedly change after being discharged four to six times, and water vapor maintained at a relatively stable level because it was exhausted quickly after it was generated. Hence, the flashover discharge voltage fluctuated in a downward trend. 

**Figure 4:**
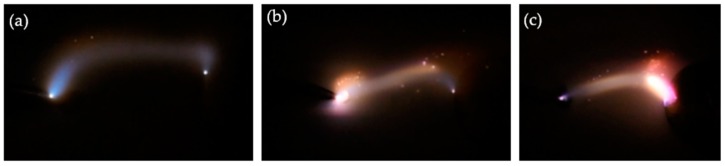
Digital images showing randomness of creepage distance at the gap distance of 9 mm.

A longer distance between the electrodes could cause a higher discharge voltage in the first three times, but longer distances cannot guarantee a higher discharge voltage as the discharge continued. In Figure 3, by keeping the distance of pin-plate electrodes at 9 mm, the flashover voltage increased slowly from 10 to 15 times flashover discharges, which can be attributed to the randomness of flashover discharge and surface structure change. A longer gap distance led to greater dispersion of discharge voltage, creating more arc discharge channels among which the actual surface creepage distance was longer than 9 mm (Figure 4). In Figure 4a, the flashover distance was obviously larger than 9 mm, while the creepage distances in Figure 4b,c were a little shorter. Mostly, the creepage distances were all above 9 mm in which the surface structure played an important role in the development direction of electrical tree. Compared with discharging at shorter gap distances, the electrical tree may develop in more directions at the gap distance of 9 mm, leading to more fluctuating flashover discharge voltages.

The surface roughness of the epoxy resin samples before flashover discharge had an average value of 0.35 μm. After 21 times discharges, there generated several visible carbonized traces, so the roughness around the traces on the samples were measured to assess the decomposition extent of epoxy resin at different gap distances. The average roughness of the epoxy resin samples after 21 times discharges was shown in Table 1.

It was reported that with the accumulation of aging energy on the material surface, the particles formed on the material surface were increased both in number and size, leading to the growth of surface roughness; and, the break of the molecular chains of epoxy resin on the surface resulted in oxidation and carbonization [25]. Increased roughness can also result in larger fluctuation of break-down voltages. Taken together, average roughness should be kept in a reasonable range for ensuring the arc creeping to be closely enough to the surface. 

### 3.2. Concentration Variation of Seven Characteristic Gases

**Figure 5:**
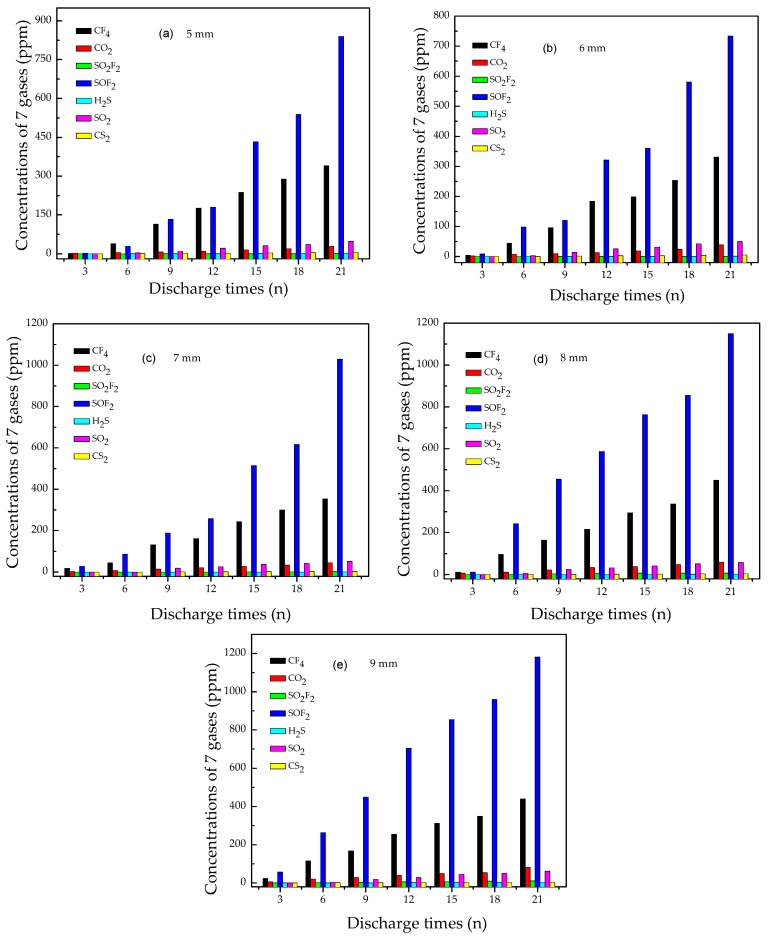
Concentration of seven gases vs. discharge times at different pin-plate electrodes distances. (**a**) 5 mm, (**b**) 6 mm, (**c**) 7 mm, (**d**) 8 mm, and (**e**) 9 mm.

Figure 5 shows the concentration of the seven characteristic gases (CF_4_, CO_2_, SO_2_F_2_, SOF_2_, H_2_S, SO_2_, and CS_2_) vs. discharge times at different pin-plate electrodes distances. The results showed a tendency that all gases concentration increased steadily with the increase of flashover discharge times. It is obvious that the concentration of SOF_2_ and CF_4_ was the highest while the concentration of other gases was relatively lower, especially SO_2_F_2_, CS_2_, and H_2_S. Taken together, the concentration of the seven characteristic gases follows the following order: SOF_2_ > CF_4_> SO_2_ > CO_2_ > SO_2_F_2_ >CS_2_ > H_2_S.

At the pin-plate electrodes distance of 5 mm, after flashover discharge six times, the concentration of SOF_2_ rose above 50 ppm, a value that can be detected easily. At the pin-plate electrodes distance of 9 mm, after flashover discharge six times, the concentration of SOF_2_ rose closely to 300 ppm. Thus, longer pin-plate electrodes distance may facilitate the generation of characteristic gas. After flashover discharge 15 times, the SOF_2_ concentration was almost twice the CF_4_ concentration, whereas other gas byproducts increased at slow rates.

#### 3.2.1. Analysis of SOF_2_ and SO_2_F_2_

**Figure 6:**
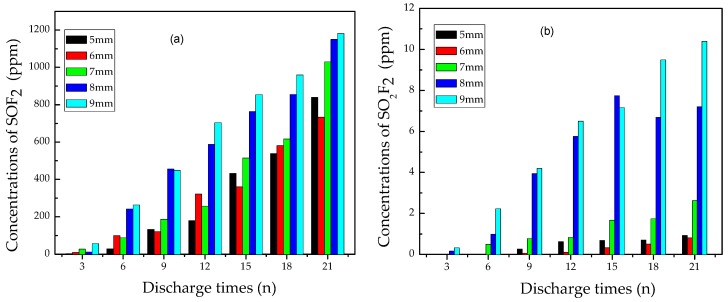
Concentration variation of (**a**) SOF_2_ and (**b**) SO_2_F_2_ vs. flashover discharge times.

The concentration of SOF_2_ and SO_2_F_2_ vs. flashover discharge times at different discharge distance were shown in Figure 6. The data indicate that SOF_2_ and SO_2_F_2_ generated steadily as the discharge times increased, and the concentration of SOF_2_ was nearly 100 times as high as that of SO_2_F_2_. After 6 times flashover discharge, SOF_2_ reached almost 300 ppm, while SO_2_F_2_ was still below 3 ppm. The concentration of SO_2_F_2_ did not reach 10 ppm before 21 discharge times, however, the SOF_2_ concentration exceeded 1000 ppm at this discharge time point. It was also found that as the discharge distance increased, the content of both SOF_2_ and SO_2_F_2_ elevated.

(1) $${\mathrm{SF}}_{2}+O\to {\mathrm{SOF}}_{2}$$

(2) $${\mathrm{SF}}_{4}+H_{2}O\to {\mathrm{SOF}}_{2}+2\mathrm{HF}$$

(3) $${\mathrm{SOF}}_{2}+O\to {\mathrm{SO}}_{2}F_{2}$$

(4) $${\mathrm{SF}}_{4}+O\to {\mathrm{SOF}}_{4}$$

(5) $${\mathrm{SOF}}_{4}+H_{2}O\to {\mathrm{SO}}_{2}F_{2}+2\mathrm{HF}$$

Dominant reaction pathways for SOF_2_ and SO_2_F_2_ were described in Equations (1)–(5). As reported previously, when partial discharge happened in SF_6_ atmosphere involving no solid insulation materials, SOF_2_ and SO_2_F_2_ concentration increased at similar rates [13,26]. However, in our experiment, the SO_2_F_2_ concentration remained far below the SOF_2_ concentration. Therefore, flashover discharge happening on epoxy resin may cause a different chemical reaction pathway to facilitate SOF_2_ generation.

Actually, under high-energy electron collision, electron-impact-induced dissociation of SF_6_ can lead to fluoride sulfide generation such as SF_5_, SF_4_, SF_3_, SF_2_, SF, and so on. It was reported that the amount of SF_4_ and SF_2_ was larger than that of other gas byproducts [26,27]. When SF_4_ comes into contact with H_2_O, SOF_2_ will soon form, as illustrated in Equation (2). SF_2_ can also be oxidized easily into SOF_2_ because S atoms of +2 valence can be turned to S atoms of +4 valence during reaction with high-active O atoms from arc discharge as shown in Equation (1). Therefore, it is reasonable to mark it as an indicator for flashover discharge fault on solid insulation surface.

Generation of SO_2_F_2_ is more complicated than that of SOF_2_. From the chemical perspective, the S atom in SOF_2_ molecule is +4 valence, that means the unsaturated chemical valence needs further oxidation to turn into SO_2_F_2_ that has an S atom of +6 valence. On the other hand, SF_4_ can be oxidized into SOF_4_, then SOF_4_ contacts with H_2_O to generate SO_2_F_2_ molecules, which is the main generation resource of SO_2_F_2_, as indicated in Equations (4) and (5) [28].

To sum up, SOF_2_ was a generated from the reaction between SF_4_ and H_2_O, while SO_2_F_2_ mainly comes from the reaction between SOF_4_ and H_2_O. SOF_4_ needs further oxidization of SF_4_ by activating O atoms. So the generation of SOF_2_ is easier than that of SO_2_F_2_. In our study, another characteristic gas CO_2_ was generated and its amount increased steadily with the increase of flashover discharge times. The generation of CO_2_ can consume a large number of active O atoms, so the SO_2_F_2_ generation became more difficult under flashover discharge happening on the epoxy resin surface, compared with partial discharge in the SF_6_ atmosphere involving no epoxy resin. As a result, the production of SO_2_F_2_ increased at a quite low rate with flashover discharge times increasing.

Without reckoning the effect of impurities such as H_2_O or O_2_ in the original SF_6_, epoxy resin decomposition may produce a small amount of water under flashover discharge, which acts as a main factor for Equation (2). Furthermore, H_2_O would decompose and release O_2_ under imposition of high-current, so Equation (1) also contributes to the generation of SOF_2_. Hence, the concentration of SOF_2_ rose rapidly as discharge continued. 

#### 3.2.2. Analysis of SO_2_

**Figure 7:**
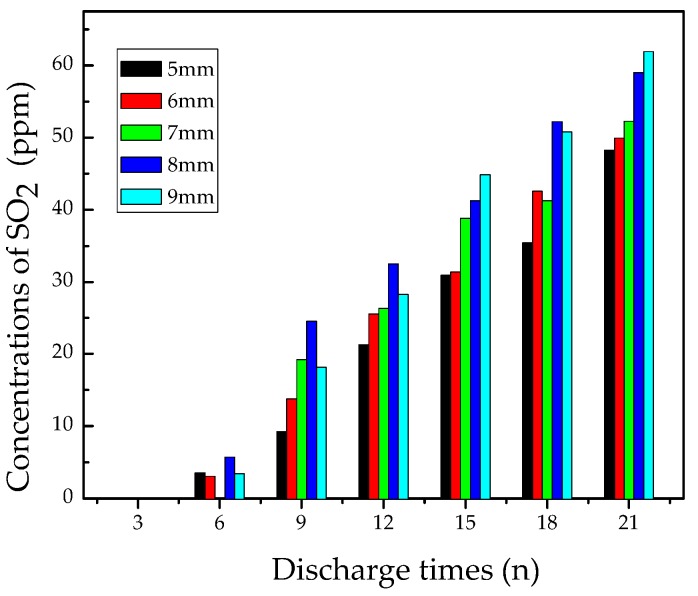
Concentration variation of SO_2_.

Figure 7 shows the concentration variation of SO_2_ vs. discharge times at different pin-plate electrodes distances. At the beginning, the amount of SO_2_ was quite small, around 5 ppm after flashover discharge six times. Later, SO_2_ was generated steadily as discharge continued, and its concentration reached to about 30 ppm and 55 ppm after discharging 12 times and 21 times, respectively. In addition, the concentration of SO_2_ also elevated with the discharge distance increasing.

S + 2O→SO_2_ (6)

SOF_2_ + H_2_O→SO_2_ + 2HF (7)

SF + OH→SO + HF(8)

SO + O→SO_2_ (9)

To better explain the concentration variation of SO_2_, the generation path of SO_2_ is depicted in Equations (6)–(9) [29]. It can be seen that the production of SO_2_ needs oxygen or water. It is extensively accepted that F atom has stronger reducibility than O atom, but the structure of SO_2_ (O=S=O) is more symmetrical than that of SOF_2_. Therefore, it is also easy for the generation of SO_2_. 

During the reactions, the amount of H_2_O was limited, and most of H_2_O reacted with subfluorides to form SOF_2_, so there is less amount of H_2_O for the generation of SO_2_ in Equation (7). Besides, there were very few S atoms provided for the Equation (6) reaction, since S atom is not the main decomposition byproduct of SF_6_. As a result, the content of SO_2_ was quite low at the beginning. As discharge continued, S and SF accumulated and had more chances to react with O_2_ and H_2_O. Thus, after about discharging nine times, the SO_2_ concentration reached a high level and increased steadily.

In conclusion, SO_2_ began to generate after a certain amount of SF_6_ decomposed into S or SF, indicating that SF_6_ has already lost its original dielectric property. In order to take SO_2_ into consideration for the flashover discharge fault diagnosis method, it is better to take the total concentration of SOF_2_ and SO_2_ as an indicator for flashover discharge fault.

#### 3.2.3. Analysis of CO_2_ and CF_4_

**Figure 8:**
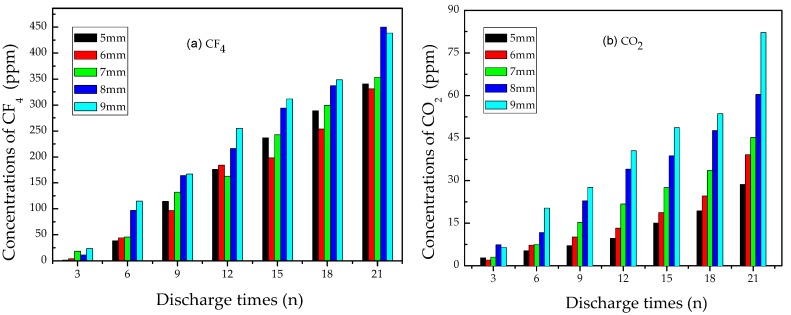
Concentration variation of (**a**) CF_4_ and (**b**) CO_2_.

Figure 8 shows the concentration variation of CO_2_ and CF_4_ vs. discharge times under different distances. Both of them were produced once the flashover discharge occurred. After discharging six times, the concentration of CO_2_ reached about 10 ppm, while CF_4_ exceeded 50 ppm. After discharging 21 times, CF_4_ concentration reached over 400 ppm, yet CO_2_ concentration was still below 90 ppm, which means that the amount of CF_4_ was nearly five times as large as that of CO_2_.

CF_4_ is an important gas byproduct involved in solid insulation materials decomposition defects. When flashover discharge occurred on epoxy resin, methyl fragments (CHx) were produced. Through substitution reaction with free F atoms, CHx would be turned into CF_4_. Flashover discharge provided enough energy for the formation of CF_4_, whereas CO_2_ mainly came from intrinsic decomposition of epoxy resin. So the concentration of CF_4_ was much higher than that of CO_2_.

In conclusion, CF_4_ has a close relationship with epoxy resin decomposition, which can indicate the occurrence of flashover discharge fault on epoxy resin dielectrics in SF_6_ insulation equipment. Therefore, the concentration of CF_4_ should be monitored intensely.

#### 3.2.4. Analysis of CS_2_

**Figure 9:**
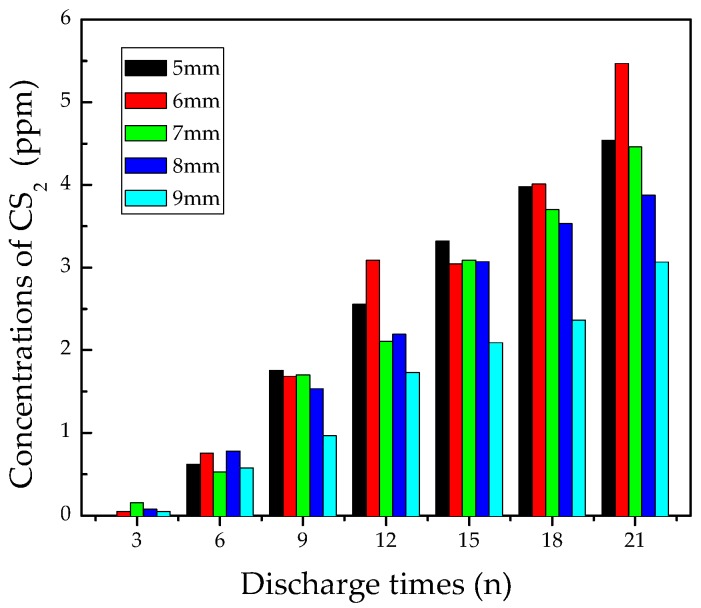
Concentration variation of CS_2_.

Figure 9 shows the concentration changes of CS_2_. It can be seen that the content of CS_2_ did not reach 3 ppm until discharging 10–15 times. As the discharge times increased, the CS_2_ amount grew slowly. After discharging 21 times, the maximum concentration of CS_2_ was still less than 6 ppm. 

The generation of CS_2_ is difficult because its generation needs active C atoms and S atoms [14,27,28]. On one hand, the amount of C atoms from the decomposition of epoxy resin was limited. The formation of SO_2_ also consumed some S atoms, so the remaining amount of S atoms from SF_6_ ionization was too low to facilitate the formation of CS_2_. C atoms mainly came from ionization of epoxy resin decomposition, and a large number of C atoms were required by the formation of CO_2_ and CF_4_, so the existing amount of CS_2_ was quite low. Besides, the 9 mm gap distance also contributed to the formation of CS_2_ to some extent.

As one of the typical byproducts of flashover discharging on epoxy resin dielectrics, CS_2_ is too difficult to be generated in comparison with CF_4_. Therefore, the appearance of CS_2_ could indicate the severest stage of flashover discharge.

#### 3.2.5. Analysis of H_2_S

**Figure 10:**
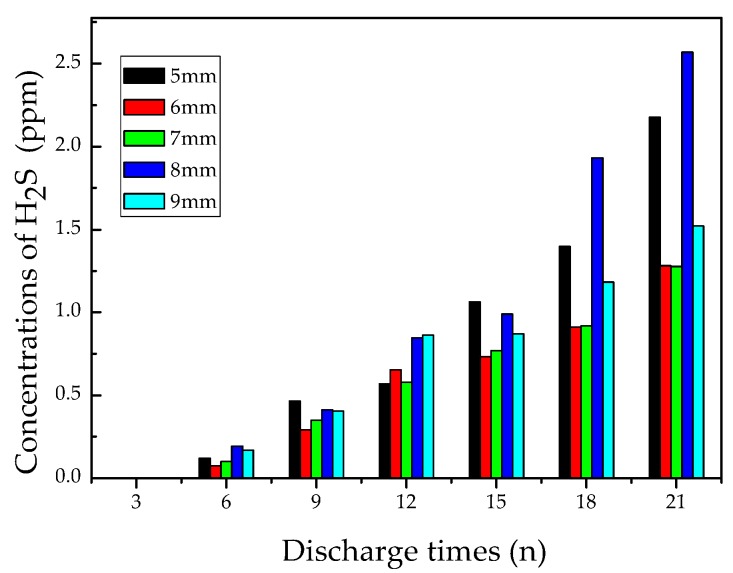
Concentration variation of H_2_S.

Figure 10 shows the concentration variation of H_2_S vs. discharge times at different pin-plate electrodes distances. The results show that the concentration of H_2_S was less than 0.3 ppm after discharging nine times, and its concentration only reached 1 ppm after discharging 15 times. After discharging 21 times, its concentration was about 2.5 ppm. A conclusion can be drawn that, like CS2, the generation of H_2_S is as difficult as that of CS_2_ among the seven characteristic gases.

The generation of H_2_S requires active S atoms and free H atoms [14,28,29]. The S atom is not the main decomposition byproduct of SF_6_ [26], and the rising concentration of SO_2_ could also reduce the reaction between S atoms and free H atoms. Therefore, the amount of H_2_S was extremely small. Although the longer distance between the electrodes can facilitate the generation of H_2_S, its amount still stayed at a very low level.

In conclusion, the generation of H_2_S can also be used as an indicator to judge the serious stage of flashover discharge faults on epoxy resin.

### 3.3. Basic Rules for Judging the Happening of Flashover Discharge

Based on the concentration variation of seven different characteristic gases above, it can be seen that SOF_2_ and CF_4_ had the highest concentration far beyond other gas byproducts, both of which can therefore represent the main decomposition component resulting from flashover discharge happening on the epoxy resin surface. Compared with the previous studies focusing on partial discharge in SF_6_ insulation equipment [1,14,17,24,29], flashover discharge on the epoxy resin surface could cause a drastically elevation in the concentration of SOF_2_ and CF_4_, but generated a relatively lower concentration for H_2_S. According to these two statistics’ data, a preliminary rule can be summarized as follows:

(10) $$c_{{\mathrm{SOF}}_{2}}>2c_{{\mathrm{CF}}_{4}}\mathrm{and}\left(c_{{\mathrm{SOF}}_{2}}+c_{{\mathrm{CF}}_{4}}\right)>400c_{H_{2}S}$$

among which, *c* represents concentration for the selected gases. That is to say, when concentration of SOF_2_ reaches twice of that of CF_4_, and the sum concentration of both SOF_2_ and CF_4_ is much higher than that of H_2_S, a possible flashover discharge on the epoxy resin surface in SF_6_-infused electrical equipment occurs. 

### 3.4. Simulation for Decomposition of Epoxy Resin under High Energy

To verify whether H_2_O mainly comes from decomposition of epoxy resin, simulation methods were employed to further investigate decomposition of epoxy resin under high energy. For the sake of simplification, the energy for epoxy resin decomposition was supplied via temperature setting. The whole simulation was conducted with the ReaxFF program and NVT ensemble. NVT represents the three parameters (number (N), volume (V), and temperature (T)) that are fixed parameters, and the other two parameters (pressure (P) and energy (E)) are variables.

**Figure 11:**
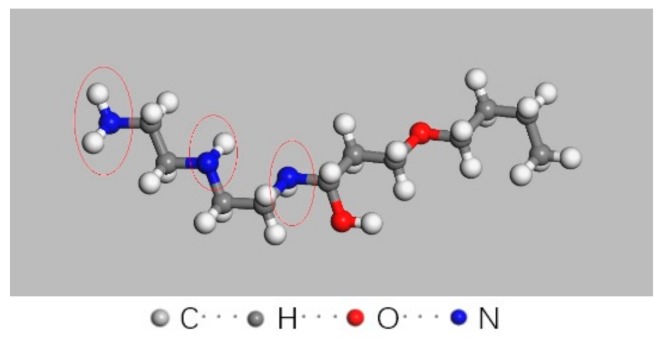
Structure of a single molecule of 593 type curing agent.

**Figure 12:**
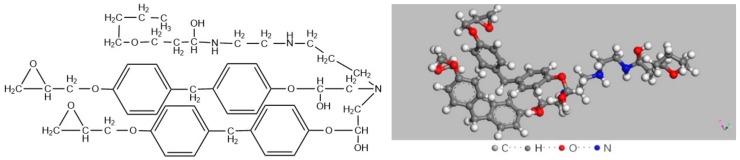
Chemical structure and ball-stick model of an epoxy resin molecule.

**Figure 13:**
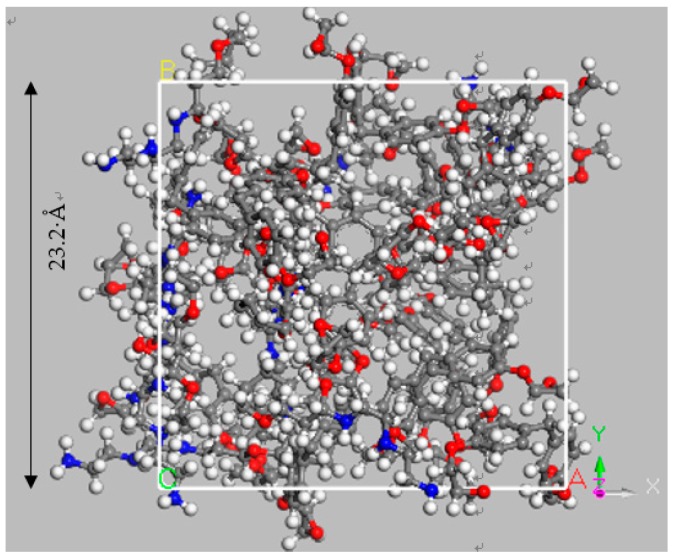
Periodic cubic box of epoxy resin molecules.

Firstly, a ball-stick model of the curing agent molecule was given in Figure 11. After curing, a bisphenol-A epoxy resin (DGEBA) molecule with polymerization degree of 0 was built, as shown in Figure 12. Then, a periodic unit cell of epoxy resin consisting of 20 epoxy resin molecules was constructed with an initial density of 0.5 g/cm^3^ in a 23.2 Å × 23.2 Å × 23.2 Å cubic box, as shown in Figure 13. The structure was treated by annealing process at the cooling rate of 10 K/500 ps and Energy Minimization and Geometry Optimization were carried out before the decomposition simulation began. The final density of the epoxy resin model was 1.13 g/cm^3^.

**Figure 14:**
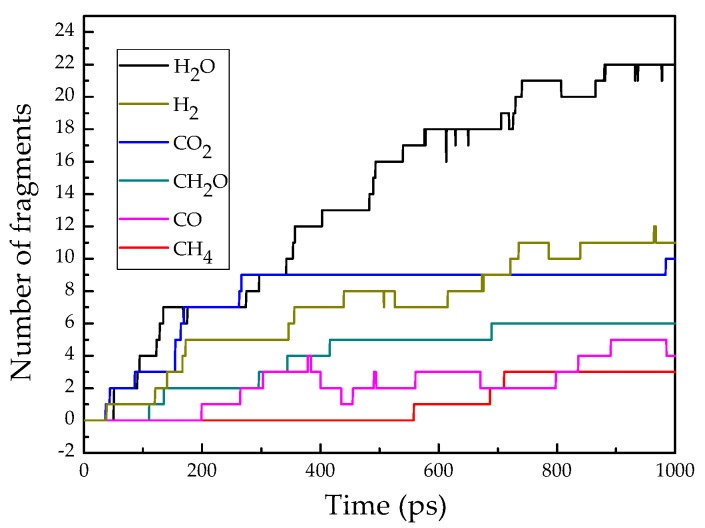
Theoretical number of fragments for the decomposition of epoxy resin heated at 1300 K over time.

**Figure 15:**
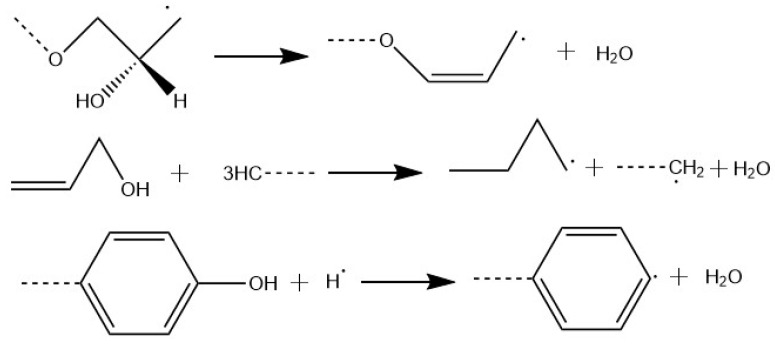
Dominant reaction pathways (RPWs) of H_2_O.

The reaction temperature was set at 1300 K to ensure that energy was high enough for epoxy resin decomposition within a total simulation time of 1000 ps [30]. It is already known that the main small molecular products are CH_2_O, H_2_O, CO, CO_2_, CH_4_, and H_2_ [30]. Figure 14 shows the number change of fragments from the periodic unit cell of epoxy resin as a function of time. It can be seen that the number of H_2_O molecules was the highest among the 6 byproducts, followed by H_2_, CO_2_, CH_2_O, CO, and CH_4_. The dominant reaction pathways (RPWs) of H_2_O were shown in Figure 15. CH_4_ was mainly derived from demethylation of methyl-group-containing fragments, and H_2_ was mainly produced by free hydrogen derived from C-H bond.

In conclusion, epoxy resin could produce H_2_O, CH_4_, CO_2_ and H_2_ that would mainly affect the concentration of seven characteristic gases. H_2_O could facilitate the formation of SOF_2_ according to Equations (1)–(3), and CH_4_ could facilitate the formation of CF_4_ through substitution reaction under high-energy arc discharge. CF_4_ decomposed from SF_6_ can react with H_2_O to form SOF_2_, leading to significantly higher concentration of SOF_2_ than other decomposition byproducts. Although the theoretical amount of CH_4_ from decomposition of epoxy resin unit cell was relatively lower, F atoms during ionization of SF_6_ can help intensify the chemical reaction changing CH_4_ to CF_4_ under arc discharge; in addition, the C-F bond in the surface structure of fluorinated epoxy resin may also break to form CF_4_ directly. Taken together, CF_4_ had the second highest concentration during flashover discharge happening on the surface of epoxy resin.

## 4. Conclusions

In the present study, flashover discharge experiments were carried out on the epoxy resin surface in SF_6_ different pin-plate electrodes distances. The concentration of seven characteristic gases including SOF_2_, SO_2_, CF_4_, CO_2_ SO_2_F_2_, CS_2_, and H_2_S were measured. The following conclusions can be drawn:

Initial flashover discharge voltage decreased sharply in the first three times of flashover discharge, and the discharge voltage fluctuated in a downward tendency.

The concentration of the seven characteristic gases elevated with the flashover discharge times increasing, and the final concentration from high to low follows the order: SOF_2_ > CF_4_ > SO_2_ > CO_2_ > SO_2_F_2_ > CS_2_ > H_2_S. Among these gases, the concentration of SOF_2_ and CF_4_ was obviously higher than that of other gases. In addition, the concentration of SO_2_F_2_, CS_2_, and H_2_S kept below 10 ppm after discharging 21 times. Based on the concentration variation of seven characteristic gases, a preliminary rule can be proposed: When the concentration of SOF_2_ reaches two times of that of CF_4_, and the sum concentration of both SOF_2_ and CF_4_ is 400 times higher than that of H_2_S, a possible flashover discharge on the epoxy resin surface in SF_6_-infused electrical equipment occurs.

The simulation for decomposition of epoxy resin on the ReaxFF program showed that epoxy resin could produce H_2_O, CH_4_, CO_2_, and H_2_ that would drastically affect the concentration of the seven characteristic gases. More specifically, H_2_O could facilitate the formation of SOF_2_, and CH_4_ could react with subfluorides to form CF_4_ under high-energy arc discharge.

## Figures and Tables

**Table 1:** Average roughness of the epoxy resin samples after 21 times flashover discharges at different pin-plate electrodes distances.

**Gap Distances (mm)**	5	6	7	8	9
**Roughness/(μm)**	0.7	0.761	0.859	0.807	0.887
